# Effect of physical activity on mortality risk among Americans with retinopathy

**DOI:** 10.15171/hpp.2016.27

**Published:** 2016-08-10

**Authors:** Paul D. Loprinzi

**Affiliations:** Jackson Heart Study Vanguard Center of Oxford, Physical Activity Epidemiology Laboratory, Center for Health Behavior Research, Department of Health, Exercise Science and Recreation Management, The University of Mississippi, University, MS 38677, USA

**Keywords:** Accelerometry, Epidemiology, Physical activity, Vision

## Abstract

**Background:** Previous work demonstrates that retinopathy is associated with increased mortality risk, with physical activity inversely associated with retinopathy and all-cause mortality. However, no study has evaluated the effects of physical activity on mortality among those with existing retinopathy, which was this study’s purpose.

**Methods:** Data from the 2005-2006 National Health and Nutrition Examination Survey were utilized, with follow-up through 2011. Retinopathy was objectively-measured using the Canon Non-Mydriatic Retinal Camera CR6-45NM. Physical activity was objectively-measured via up to 7 days of accelerometry assessment.

**Results:** Six-hundred and seventy one adults (40-85 years) with complete data on the study variables constituted the analytic sample. During the follow-up period, 91 deaths occurred. In the sample, 35 886 person-months occurred with a mortality incidence rate of 2.5 deaths per1000 person-months. Among participants with mild retinopathy, those who met physical activity guidelines at baseline had a 63% reduced risk of all-cause mortality (HR _adjusted_ = 0.37; 95% CI:0.18-0.75; P = 0.007). Notably, physical activity was not associated with mortality risk among those with moderate/severe retinopathy (HR _adjusted _= 0.371.72; 95% CI: 0.62-4.76; P = 0.27).

**Conclusion:** Physical activity is associated with reduced mortality risk among those with mild retinopathy, but not among those with moderate/severe retinopathy.

## Introduction

Retinopathy is associated with increased mortality risk.^1^ The mechanisms through which retinopathy may increase mortality risk is not fully elucidated. It is possible that retinopathy increases mortality risk through shared risk factors. For example, cardiovascular and mortality risk factors, such as hypertension, dyslipidemia and elevated glycated hemoglobin, are known to increase the risk of retinopathy.^2,3^ Our recent work demonstrates that physical activity is associated with reduced retinopathy prevalence,^2^ reduced mortality risk among those with visual impairment,^4^ and reduced mortality risk among those at high risk for a future atherosclerotic cardiovascular disease event.^5^ Yet to be investigated in the literature, however, is whether physical activity plays a protective role against mortality risk among those with existing retinopathy, which was this study’s purpose, written here as a short communication. The hypothesis is that physical activity will be inversely associated with mortality risk even among those with existing retinopathy. This hypothesis is plausible as previous research demonstrates that physical activity is favorably associated with both retinopathy and mortality risk.^2,5^ If indeed physical activity plays a protective role in mortality risk among those with existing retinopathy, then this will have important health promotion implications for those with retinopathy.

## Materials and Methods

### Design & Participants

Data from the 2005-2006 National Health and Nutrition Examination Survey were used. In this sample, 671 adults (40-85 years) with complete data on the study variables constituted the analytic sample. Only those with some degree of retinopathy were evaluated herein (N=566, mild retinopathy; N=105, moderate/severe retinopathy).

### Retinopathy

As we have described elsewhere,^2^ retinal imaging was performed using the Canon Non-Mydriatic Retinal Camera CR6-45NM (Canon, Tokyo, Japan). The presence of non-proliferative retinopathy (mild or moderate/severe retinopathy) was determined using the Early Treatment Diabetic Retinopathy Study grading criteria.^6^

### Physical activity

As described and validated elsewhere,^7^ self-reported physical activity was assessed. Consistent with government physical activity guidelines (30 min/day of moderate-to-vigorous physical activity [MVPA]), participants were classified as above or below 2000 MVPA MET-min-month.

### Statistical analyses

All analyses were performed using survey data procedures to adjust for the complex survey design employed in NHANES. Multivariable Cox proportion hazard analysis was employed to examine the association of meeting physical activity guidelines and mortality, with analyses stratified by those with mild retinopathy and moderate/severe retinopathy. Hazard ratios (and their corresponding 95% confidence interval) were calculated as an estimate of an effect size. Schoenfeld’s residuals were used to verify the proportional hazards assumption. Analyses were adjusted for age, gender, race-ethnicity, diabetes (physician diagnosis, A1C≥6.5%, or fasting glucose ≥126 mg/dL), objectively-measured visual impairment (normal, URE, or VI),^2^ and comorbid illness (summed number of the following conditions: coronary artery disease, stroke, heart attack, body mass index (BMI) ≥ 25 kg/m^2^, hypertension diagnosis, and cancer diagnosis).Statistical significance was established as *P*<0.05, with all analyses evaluated in the Stata software package (v. 12, College Station, TX).

## Results

Characteristics of the analyzed sample are shown in Table 1. Participants, on average, were 59.8 (95% CI: 58.2-61.5) years, and the mean number of comorbidities was just over 1 for the entire sample. Among those with mild and moderate/severe retinopathy, respectively, approximately 56% and 50% were men. A larger percentage of individuals with mild retinopathy were non-Hispanic white when compared to those with moderate/severe retinopathy (70.5% vs. 52.9%). As expected, diabetes was more prevalent among those with moderate/severe vs. mild retinopathy (88.8% vs. 33.7%). Further, visual impairment was also more prevalent among those with moderate/severe (7.5%; 95% CI: 0.9-14.1) vs. mild retinopathy (1.7%; 95% CI: 0.4-2.9).

The median follow-up period was 55 months (IQR=43-66). During the follow-up period, 91 deaths occurred. In the sample, 35886 person-months occurred with a mortality incidence rate of 2.5 deaths per 1000 person-months. Among participants with mild retinopathy, those who met physical activity guidelines at baseline had a 63% reduced risk of all-cause mortality (HR_adjusted_ = 0.37; 95% CI: 0.18-0.75; *P*=0.007); proportional hazard assumption was not violated (*P*=0.85). Notably, physical activity was not associated with mortality risk among those with moderate/severe retinopathy (HR_adjusted_ = 1.72; 95% CI: 0.62-4.76; *P*=0.27); proportional hazard assumption was not violated (*P*=0.40) (Table 2).

**Table 1:** Characteristics of the analyzed sample (N = 671)

	**Mild retinopathy**	**Moderate/severe retinopathy**
N	566	105
Age, mean (95% CI) years	59.8 (58.1-61.6)	59.8 (57.3-62.3)
Comorbidities, mean (95% CI)	1.2 (1.1-1.4)	1.6 (1.4-1.9)
Men, %	55.8	49.9
White, %	70.5	52.9
Diabetes, %	33.7	88.8
Visual impairment, %	1.7	7.5
Died, %	10.2	17.5
Meeting PA guidelines,^a^ %	16.4	20.0

Abbreviations: PA, physical activity; MVPA, moderate-to-vigorous physical activity. ^a^ Meeting PA guidelines defined as ≥2000 MVPA MET-min-month.

**Table 2:** Weighted multivariable Cox proportional hazard model evaluating the association between physical activity and mortality risk, stratified by retinopathy status

	**Mild Retinopathy**		**Moderate/Severe Retinopathy**	
	**HR**	**95% CI**	**HR**	**95% CI**
Meeting MVPA guidelines vs. not	0.37	0.18-0.75	1.72	0.62-4.76

Abbreviations: MVPA, Moderate-to-vigorous physical activity; HR, hazard ratio. Meeting PA guidelines defined as ≥ 2000 MVPA MET-min-month. Models adjusted for age, gender, race-ethnicity, diabetes, visual impairment and comorbid illness.

## Discussion

Adults with retinopathy have an increased risk of early mortality.^1,8-13^ For example, in a meta-analysis of 20 studies, providing data from 19234 patients, Kramer et al^1^ demonstrated that, among those with type 2 diabetes, the presence of any degree of diabetic retinopathy increased the chance for all-cause mortality and/or cardiovascular disease events by 2.34. Results were similar for adults with type 1 diabetes. Consequently, these findings highlight that diabetic retinopathy, in particular, is a serious microvascular complication. As such, individuals with retinopathy should be carefully screened and monitored, along with the provision of necessary resources to mitigate cardiovascular disease and mortality risk.

Encouragingly, the present findings demonstrate that physically activity adults with mild retinopathy have a reduced risk of all-cause mortality. This finding is in alignment with other studies showing that physical activity is protective of early mortality among various vulnerable populations, such as those with a high risk for cardiovascular disease,^5^ coronary artery disease patients,^14,15^ congestive heart failure patients,^16,17^ diabetics,^18^ hypertensive adults,^19^ chronic obstructive pulmonary disease patients,^20^ liver disease patients,^21^ and those with visual^4^ or hearing impairment.^22,23^ Physical activity, however, was not protective of early mortality among those with moderate/severe retinopathy, which may be a result of the greater degree of visual impairment (which is linked with reduced activity and increased mortality risk) among this group. As such, these findings underscore the importance of physical activity promotion among those with varying degrees of retinopathy, but particularly before the progression from mild to moderate retinopathy.

Major strengths of this study include the novel investigation, national sample and prospective study design. Future replicative work is needed, which should overcome the limitations of this study, including the subjective assessment of physical activity and relatively short follow-up period.

In conclusion, the results of the present study suggest that physical activity is inversely associated with mortality risk among those with mild retinopathy, but not among those with moderate/severe retinopathy. Future confirmatory work is needed, and such work would benefit by evaluating candidate mechanisms to explain the present study’s observed associations.

## Funding

No funding was used to prepare this manuscript.

## Ethical approval

Procedures were approved by the National Center for Health Statistics review board; written consent was obtained prior to data collection.

## Competing interests

The authors declare no conflicts of interest.

## Author’s contribution

PDL was involved in the conceptualization of the study, data analyses, interpretation of the results, and drafting and revising the manuscript.
